# Improving Surgical Scene Semantic Segmentation through a Deep Learning Architecture with Attention to Class Imbalance

**DOI:** 10.3390/biomedicines12061309

**Published:** 2024-06-13

**Authors:** Claudio Urrea, Yainet Garcia-Garcia, John Kern

**Affiliations:** Electrical Engineering Department, Faculty of Engineering, University of Santiago of Chile, Las Sophoras 165, Estación Central, Santiago 9170020, Chile; yainet.garcia@usach.cl (Y.G.-G.); john.kern@usach.cl (J.K.)

**Keywords:** deep learning for laparoscopic surgery, class imbalance, activation function, loss function, Adam and SGDM optimizers, semantic segmentation of the surgical scene

## Abstract

This article addresses the semantic segmentation of laparoscopic surgery images, placing special emphasis on the segmentation of structures with a smaller number of observations. As a result of this study, adjustment parameters are proposed for deep neural network architectures, enabling a robust segmentation of all structures in the surgical scene. The U-Net architecture with five encoder–decoders (U-Net5ed), SegNet-VGG19, and DeepLabv3+ employing different backbones are implemented. Three main experiments are conducted, working with Rectified Linear Unit (ReLU), Gaussian Error Linear Unit (GELU), and Swish activation functions. The applied loss functions include Cross Entropy (CE), Focal Loss (FL), Tversky Loss (TL), Dice Loss (DiL), Cross Entropy Dice Loss (CEDL), and Cross Entropy Tversky Loss (CETL). The performance of Stochastic Gradient Descent with momentum (SGDM) and Adaptive Moment Estimation (Adam) optimizers is compared. It is qualitatively and quantitatively confirmed that DeepLabv3+ and U-Net5ed architectures yield the best results. The DeepLabv3+ architecture with the ResNet-50 backbone, Swish activation function, and CETL loss function reports a Mean Accuracy (MAcc) of 0.976 and Mean Intersection over Union (MIoU) of 0.977. The semantic segmentation of structures with a smaller number of observations, such as the hepatic vein, cystic duct, Liver Ligament, and blood, verifies that the obtained results are very competitive and promising compared to the consulted literature. The proposed selected parameters were validated in the YOLOv9 architecture, which showed an improvement in semantic segmentation compared to the results obtained with the original architecture.

## 1. Introduction

The segmentation of surgical images is fundamental for disease treatment and diagnoses, computer-assisted surgery, and surgical robot control, planning, and navigation. In the case of abdominal surgeries, organ segmentation is decisive, and multiorgan segmentation is desirable in digital images or real-time videos. Abdominal organs present a great variety in terms of size and shape. There are also interrelationships among them, and even the occurrence of abnormalities in anatomic structures and/or the presence of anomalous structures. All these situations, added to the complexity of the background and blurry limits, make multiorgan segmentation a challenging task.

The segmentation of structures based on traditional methods uses, in general, characteristics of the image, for example, intensity, borders, and gradients, among others, to apply techniques such as thresholding and region-growing. However, some factors make them not robust enough for this task, for example, dependence on the selection of threshold values, lighting problems, and the intrinsic characteristics of such structures. These methods are only used for approximated organ segmentation or specific organ segmentation [1,2,3].

Artificial Intelligence (AI) has improved the task of identifying anatomy in the surgical field. In this sense, deep learning (DL) achieves great accuracy in segmentation, especially in semantic segmentation, which enables the segmentation of multiple objects in the scene. Semantic segmentation offers advantages in terms of segmentation speed and adaptability to different organs, which is useful for medical applications and even promotes real-time intrasurgical guidance [1,4].

### Related Work

**Figure 1:**
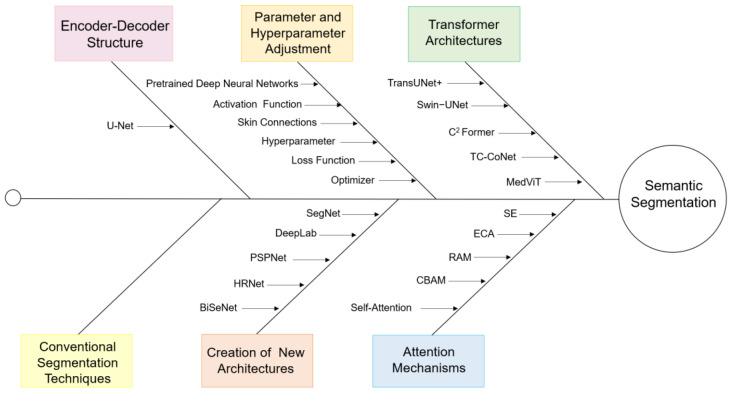
Milestones in semantic segmentation evolution.

Semantic segmentation is a typical task of computer vision that represents a classification task at the pixel level. This task aims to understand the role of each pixel in the image, for which it creates pixel groups that can later be labeled and classified. With this process, each object can be delimited, obtaining segmentation masks coded by pixel color for the classified objects [5,6,7]. There is a wide variety of algorithms for semantic segmentation in medical images; however, this task has been enriched and dominated by DL techniques. The U-Net architecture is widely recognized in the medical field and stands out for popularizing the use of the encoder–decoder configuration, thus marking a milestone in the development and evolution of semantic segmentation. Figure 1 summarizes some of the key milestones that have marked the evolution of semantic segmentation in medical images.

As is evident, U-Net inspired the emergence and development of other architectures based on the encoder–decoder configuration, such as SegNet, DeepLab, and Pyramid Scene Parsing Network (PSPNet) [8,9,10]. Another significant contribution to the efficiency of semantic segmentation involves the use of different optimizers, the tuning of hyperparameters, the implementation of skip connections, and the appropriate selection of the activation function, among others.

The emergence of attention mechanisms represented a significant advancement, as they allow for greater focus on features of interest while disregarding less relevant ones based on the study’s specific objectives [11]. Among the most well-known attention modules are Squeeze-and-Excitation (SE), Convolutional Block Attention Module (CBAM), Efficient Channel Attention (ECA), and Residual Attention Module (RAM), among others [12,13,14,15]. The self-attention mechanism stands out for its performance and is crucial for the introduction of the Transformer architecture [16].

Given these advances, and particularly in the field of medical images, several studies have used U-Net as a starting point to develop new architectures. For instance, in [17], a combination of a convolutional neural network (CNN) and a Transformer is employed to extract local and global features. Additionally, the Three-Level Attention (TLA) attention module is applied, along with skip connections with a distribution similar to UNet++ [18]. Li et al. [19] implemented IB-TransUNet, merging the Information Bottleneck and Transformer into the U-Net model, and in [20], they proposed the MultiIB-TransUNet architecture. Some more recent architectures include High Correlative Non-Local Network (HCNNet), Bilateral Segmentation Network (BiSeNet V3), Contoured Convolutional Transformer (CCTrans), Cross-Convolutional Transformer Network (C^2^Former), Double-stage Codec Attention Network (DSCA-Net), and Medical Vision Transformer (MedViT) [21,22,23,24,25,26]. Additionally, specific architectures have been designed for the processing of 3D medical images [27].

The semantic segmentation of structures in the abdomen using RGB or RGB-D images is implemented in [4,28,29,30], where work is carried out with frames obtained from laparoscopic cholecystectomy videos. In [31,32,33], images from the CholecSeg8K dataset are used; however, these works do not consider the success rate for structures with a lower number of samples or appearances. An important aspect that requires attention in the semantic segmentation of RGB images of abdominal laparoscopic surgery is class imbalance, which is a critical point in working with medical images in general.

Approaches to address class imbalance can be divided into three main groups: data-level approaches, where undersampling of the majority class and oversampling of the minority class stand out; algorithm-level approaches; and ensemble learning approaches [33,34]. To address this, ref. [35] proposes data augmentation by creating synthetic images using a Generative Adversarial Network (GAN). In this regard, Hassanat, A. B. et al. suggest in [33,34] that synthesizing new instances without proper precautions could have serious repercussions in the medical field. Consequently, they propose the use of ensemble approaches, random data partitioning, and method-level approaches as alternatives to oversampling, as they lack erroneous assumptions. On the other hand, in [36,37], loss functions and other adjustments are used to improve the texture of the edges of small structures.

The following article focuses on the segmentation of the surgical scene in RGB images, for which various DL architectures are implemented. The main objective is to analyze and evaluate the effect of different parameters on the performance of semantic segmentation. The success rate is evaluated, paying special attention to the semantic segmentation of small structures or those with a lower number of appearances in the dataset, which have not been considered in the literature consulted. In this regard, the main contributions of this study are the following:

- Improvement in the performance of DL architectures in intraoperative semantic segmentation. The proposed parameter configuration, in terms of the optimizer, activation function, and loss function, among others, allowed for an accurate semantic segmentation of the surgical scene in abdominal laparoscopic surgery images, achieving the best overall performance in the consulted literature.
- An efficient approach to class imbalance, achieving high success rates in the semantic segmentation of structures such as the hepatic vein, cystic duct, Liver Ligament, and blood, which have been little analyzed in the state of the art due to their small dimensions and/or lower number of annotations in the dataset under study.
- The adaptation of the YOLOv9 architecture for semantic segmentation of the surgical scene. With the parameter configuration proposed in this study, the performance of the architecture on the dataset under study is improved, compared to the results obtained with its original configuration.

This manuscript comprises five sections. Section 1 contextualizes the needs of studying and promoting the semantic segmentation of the surgical scene in RGB images for the development and evolution of robotic surgery, among other applications. Section 2 describes the methodology followed, as well as the design parameters considered. Section 3 presents the results obtained and a comparative analysis of them is conducted. In Section 4, the results, clinical value, main findings, and limitations of the research are discussed. Lastly, Section 5 presents the conclusions of the experiment and future work is addressed.

## 2. Materials and Methods

In this study, known DL architectures are implemented, which have often served as the basis for the development of new architectures or the improvement in older architectures. The performed analysis is based on three main experiments, in which diverse parameters were adjusted for the design of DL architectures employed in the semantic segmentation of laparoscopic surgery images. This study used MatLab^®^ R2023a software, a Lenovo AMD Ryzen 9 5900HX 3.3 GHz laptop with 32 GB RAM, and a GPU NVIDIA GeForce RTX 3080 card as the hardware.

### 2.1. Dataset

**Figure 2:**
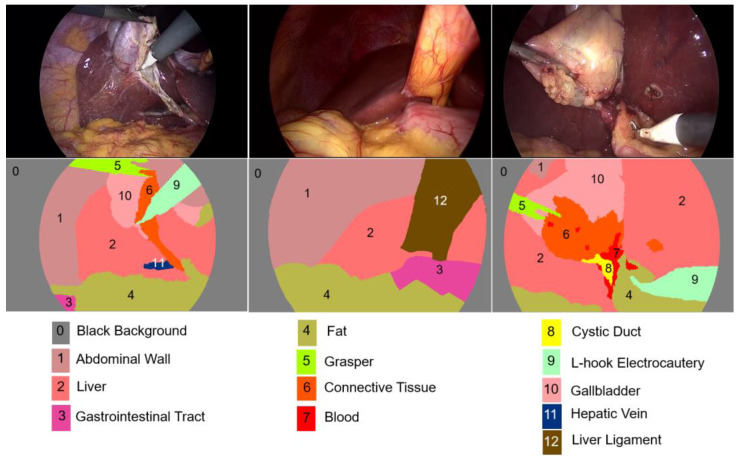
Images from the CholecSeg8K dataset.

The images employed for the training and testing of neural networks belong to the CholecSeg8K dataset [38,39]. The CholecSeg8K dataset, derived from the Cholec80 database [40], contains a subset of laparoscopic cholecystectomy surgery videos performed by 13 surgeons. Figure 2 shows examples of RGB images from the dataset. The first row displays the input images, while the second row presents the ground truth of their semantic segmentation, which is representative of all the classes present in the dataset.

CholecSeg8K contains 8080 frames with a resolution of 854 × 480 in Portable Network Graphics (PNG) format. Each image is annotated at the pixel level for thirteen common classes in laparoscopic cholecystectomy surgery. It is important to note that not all 13 classes are shown simultaneously in each frame. For each frame, the dataset includes three types of masks: a hand-drawn mask during annotation using the Pixel Annotation Tool [41], a color mask for better visualization, and a watershed mask that facilitates algorithmic processing. This dataset has been made publicly available under the CC BY-NC-SA 4.0 license.

### 2.2. DL Architecture for Semantic Segmentation

#### 2.2.1. U-Net5ed Architecture

**Figure 3:**
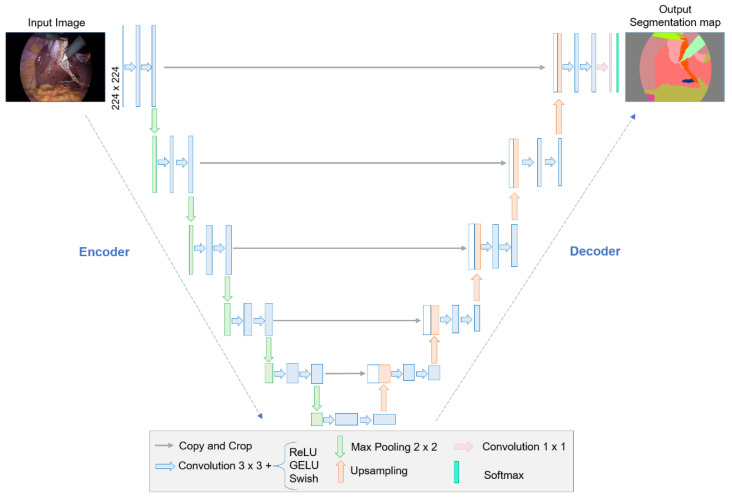
U-Net5ed architecture with five encoder–decoders.

The U-Net architecture was proposed in 2015 in [42] for the segmentation of biomedical images as a promising alternative to obtain satisfactory segmentations through a small set of training images. The structure of this neural network has a U shape that follows a contraction and expansion route from left to right, respectively. The U-Net is characterized by skip connections that transport the information from the encoding to the decoding layers. Considering the general U-Net structure presented in [42], Figure 3 shows the adaptation made to it for the same characteristics for the following study, in which five encoder–decoders (U-Net5ed) are implemented.

During encoding, the information contained in the input image is compressed into feature maps with lower resolution but more semantic information. This is caused by the presence of convolutional and pooling layers that, while reducing their size, allow for the extraction of features and details from the image at different levels of abstraction, to capture its context and relevant information. Meanwhile, decoding represents the opposite process to encoding. To conduct encoding, the lower-resolution feature maps obtained during encoding are subject to upsampling and deconvolution layers that increase their size until obtaining an output with the same resolution as the input image. During this process, the semantic information is combined with the spatial details to generate the final segmentation of the image [43].

#### 2.2.2. SegNet Architecture

**Figure 4:**
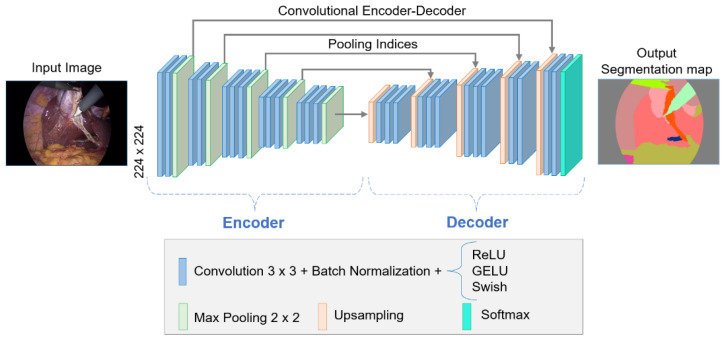
SegNet architecture.

SegNet is an encoder–decoder convolutional neural network for the semantic segmentation of images. This architecture was mainly designed for scene understanding applications, and therefore its design aims to be efficient in terms of memory and calculation time. However, the number of trainable parameters in it is smaller than in other architectures. Considering the general structure of the SegNet network presented in [9], Figure 4 shows its adaptation for the following study.

#### 2.2.3. DeepLabv3+ Architecture

**Figure 5:**
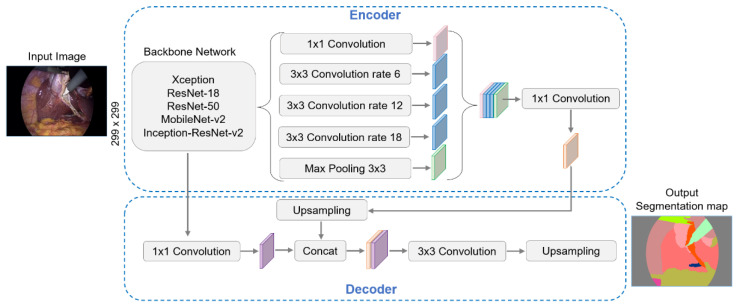
DeepLabv3+ architecture.

DeepLab models are very popular and have offered good results in various applications. They employ an encoder–decoder architecture that uses atrous separable convolution. The DeepLabv3+ version is an improvement over previous versions. Considering the general structure of this network presented in [44,45], Figure 5 shows its adaptation for the following study.

#### 2.2.4. Pretrained Deep Neural Networks

Pretrained neural networks have already learned to extract representative and informative features from images, and therefore it is efficient to use them as a starting point for learning a new task through transfer learning, which is faster and easier than training a network from scratch. Most pretrained networks are trained with a subset of the ImageNet database. These networks have been trained with more than one million images and can classify them into 1000 object categories. When selecting one of these networks, factors such as their accuracy, speed, and network size should be considered. Some of the pretrained networks that are most commonly and widely used in medical images are ResNet-18, MobileNet-v2, and Inception-ResNet-v2, among others [46].

### 2.3. Training Parameters

There is a set of adjustment parameters that directly affect the training of a network and the performance of the semantic segmentation model. This is the case with hyperparameters. Their adequate selection depends on several factors such as the complexity of the specific situation, the characteristics of the dataset, the DL architecture to be used, and the optimizer. At the same time, the activation and loss functions are decisive in learning, and thus their selection should be conducted carefully, considering all the characteristics of the problem.

There are several optimizers that present adequate results with learning algorithms that are iterative and based on the gradient, for which the value of the cost function is reduced to a lower value conditioned by the non-linearity of the neural network. Among the most popular optimizers are Stochastic Gradient Descent (SGD), SGD with momentum (SGDM), and Adaptive Moment Estimation (Adam). SGD is an optimization algorithm applicable to different problems, although its use reports some degree of instability in the optimization or learning process.

In the case of SGDM, momentum can soften the progression of the learning algorithm in the training process. A momentum with 0.9 and 0.99 values allows for reaching reasonable accuracy values during the training of approximately 50 epochs, compared to the need for implementing 200 epochs when this parameter does not apply. The Adam optimizer was proposed in [47]; this is a computationally efficient and easy to implement algorithm that stands out due to its robust behavior in terms of parameter selection [48,49]. In this study, the results offered by SGDM and Adam are considered for semantic segmentation.

#### 2.3.1. Activation Function

Using linear activation functions limits the capacity of a neural network to recognize patterns and make accurate predictions, hence the use of non-linear activation functions. The most renowned and widespread activation function in the output layer is Softmax. In semantic segmentation, this function allows for generating a probability distribution for each pixel of the input image. Therefore, Softmax promotes the generation of a segmentation mask where each pixel is assigned the class with the highest probability.

The most known and widely used activation function in intermediate layers is Rectified Linear Unit (ReLU), among others, such as the tangent and sigmoidal activation functions. There are other activation functions that, despite reports in the literature that they involve an increase in computing cost, may offer superior results. The activation function of Gaussian Error Linear Unit (GELU) proposed in [50] has been employed by several Transformer architectures. Some other functions exhibit very promising results, for example, Swish and Mish [51,52]. In this study, the operation of ReLU, GELU, and Swish will be measured, which are defined in Equations (1), (2), and (4), respectively [46]. A ReLU layer conducts a threshold operation for each input element, setting to zero any element that has a value smaller than zero. Meanwhile, GELU weighs the input by its probability under a Gaussian distribution.

(1) $$RE\left(x\right)=\left\{\begin{array}{c} x,\;\;x\geq 0\\ 0,\;\;x<0 \end{array}\right.$$

(2) $$GE\left(x\right)=\frac{x}{2}\left(1+erf\left(\frac{x}{\sqrt{2}}\right)\right)$$

where the term RE denotes the ReLU activation function, the term GE denotes the GELU activation function, and erf denotes the error function defined as Equation (3).

(3) $$erf\left(x\right)=\frac{2}{\sqrt{\pi }}\int_{0}^{x}e^{-t^{2}}dt$$

(4) $$SW\left(x\right)=\frac{x}{1-e^{-x}}$$

where the term SW denotes the Swish activation function.

#### 2.3.2. Loss Function

**Figure 6:**
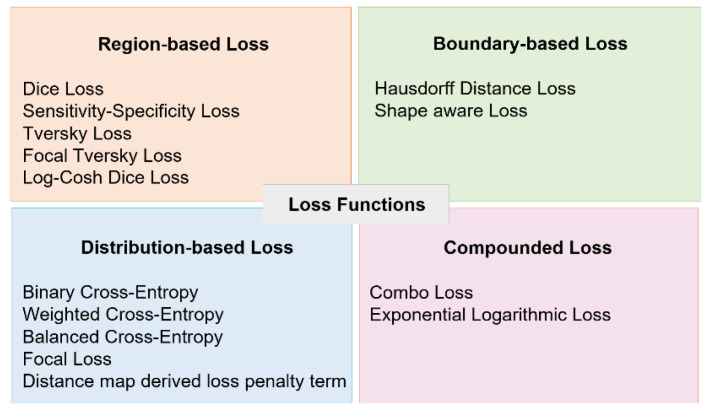
Loss functions employed in segmentation.

The loss function influences the success of neural network learning and is directly related to the application to be developed. In [53], four categories for loss function are proposed, whose results are relevant to different segmentation applications, which are shown in Figure 6. In general, the selection of the loss function should consider, among other aspects, the types and quantity of classes, as well as the quantity of pixels in each class, especially under data imbalance. In classification models based on images and patches, the Cross Entropy (CE) loss function is often used. In the case of pixel-based classification models, the CE and Dice Loss (DiL) functions are used more frequently, as well as variants of these that include their combination.

The Focal Loss (FL) loss function proposed in [54] reports a good performance in the presence of classes with few observations. This function consists of introducing a modulating factor into the CE standard criterion, which reduces the relative loss of well-classified pixels and increases the sensitivity of the network to incorrectly classified pixels. Equations (5) and (6) define the CE and FL loss functions, respectively, which will be used in this study.

(5) $$CE\left(p_{t}\right)=-\log p_{t}$$

(6) $$FL\left(p_{t}\right)=-\alpha_{t}{\left(1-p_{t}\right)}^{\gamma }\log p_{t}$$

where the term CE denotes the Cross-Entropy Loss function, the term FL denotes the Focal Loss function, and the term $p_{t}$ represents the estimated probability for a specific pixel based on its belonging to the target semantic class, defined in Equation (7). The term $\alpha_{t}$ defined in Equation (8) represents an imbalance factor that adjusts the importance of positive and negative examples. Its inclusion may slightly improve the accuracy of the segmentation process.

The expression 1−p represents the estimated probability that the pixel belongs to any other semantic class that is not the target class. The expression ${\left(1-p_{t}\right)}^{\gamma }$ proposed in [54] controls the contrast of loss value; parameter γ denotes a prefixed positive scale value that can be γ≥0. This expression acts as a modulating factor, reducing the weight of easy examples (with high estimated probabilities $p_{t}$, which makes them easier to be correctly classified) and focusing on the harder examples during the training of the semantic segmentation model. If γ=0, FL equals the loss of CE and the larger γ becomes, the larger the contrast between loss values of easy and hard classes becomes [54,55,56].

(7) $$p_{t}=\left\{\begin{array}{l} p\;\;\;\;\;\;\;\;\;\mathrm{if}\;\;\;\;\;\;\;\;y=1\\ 1-p\;\;\;\mathrm{otherwise} \end{array}\right.$$

(8) $$\alpha_{t}=\left\{\begin{array}{l} \alpha \;\;\;\;\;\;\;\;\;\mathrm{if}\;\;\;\;\;\;\;\;y=1\\ 1-\alpha \;\;\;\mathrm{otherwise} \end{array}\right.$$

where y∈±1 (specifies the ground-truth class), p ∈ [0, 1] represents the probability estimated by the model of the pixel belonging to the target semantic class with the label y=1, and α ∈ [0, 1] is a weighting factor.

The Tversky Loss (TL) function has also been employed to reduce the effect of class imbalance and applied in works with medical images. This function is an asymmetric measure and consists of a generalization of the Jaccard or Intersection over Union (IoU) index, and the Dice index. TL was proposed in [57], and is inspired by the Tversky index (${TI}_{c}$). Equation (9) defines ${TI}_{c}$ between an image (J) and its ground truth (G).

(9) $${TI}_{c}=\frac{\sum_{m=1}^{M}J_{cm}G_{cm}}{\sum_{m=1}^{M}J_{cm}G_{cm}+\sigma \sum_{m=1}^{M}J_{cm}G_{\bar{c}m}+\beta \sum_{m=1}^{M}J_{\bar{c}m}G_{cm}}$$

where the class is represented by c and $\bar{c}$ corresponds to not being in the class; M represents the number of elements in the first two dimensions of J, while σ and β weigh the contribution of False Positives (FPs) and False Negatives (FNs) to the loss, respectively. TL is defined in (10) as a function of C classes [46]. When σ=β=0.5, the DiL loss function is being used.

(10) $$TL=\sum_{c=1}^{C}1-{TI}_{c}$$

where C represents the number of classes, the term TL denotes the Tversky Loss function, and the term ${TI}_{c}$ denotes the Tversky index.

The use of combined or hybrid loss functions is growing in many applications. These modifications are made with the main goal of improving DL models. In this study, the combination of the loss functions CE and DiL, denominated Cross Entropy Dice Loss (CEDL), and of CE and TL, denominated Cross Entropy Tversky Loss (CETL), was assessed. The combinations are represented in Equations (11) and (12), respectively, in which the parameter δ is introduced to give higher priority to one of the combined loss functions.

(11) $$CEDL=\delta \cdot CE+\left(1-\delta \right)\cdot DiL$$

(12) $$CETL=\delta \cdot CE+\left(1-\delta \right)\cdot TL$$

where the parameter δ is introduced to give higher priority to one of the combined loss functions.

### 2.4. Experiment Conducted

**Figure 7:**
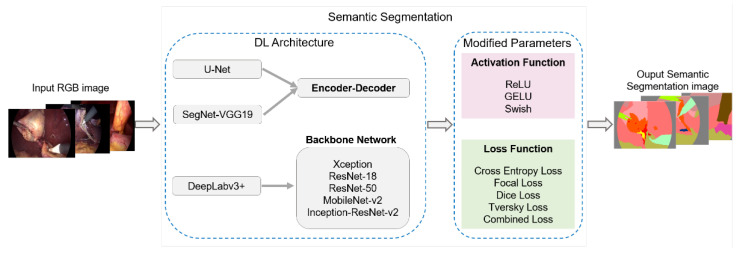
Block diagram of experiment.

The following experiment is conducted with the main goal of testing the performance of the DL architectures selected for this study, as well as the effects of the adjustment of different parameters on the quality of semantic segmentation. The experiment will focus on the success of the segmentation of structures with a low number of segmentations. This analysis will allow for creating a DL structure that enables the robust segmentation of structures in the surgical scene. Figure 7 describes the methodology followed, which employs the U-Net, SegNet, and DeepLabv3+ architectures.

In the case of the first two DL architectures, the number of encoder–decoders to use is selected. For DeepLabv3+, the pretrained neural networks Xception, ResNet-18, ResNet-50, MobileNet-v2, and Inception-ResNet-v2 are used as the backbone. Then, the parameters of the activation function and the loss function of each configuration are modified to test their performance.

### 2.5. Metrics

Among the main metrics used for comparing the quality of segmentation methods, accuracy (Acc) is represented in Equation (13). The analysis of other metrics will be conducted, such as Intersection over Union, the boundary F1 contour matching score for image segmentation (bfs), and the Dice similarity coefficient (Dice), represented in Equations (14)–(16), respectively [53,57].

(13) $$Acc=\frac{TP+TN}{TP+TN+FP+FN}$$

(14) $$IoU=\frac{TP}{TP+FP+FN}$$

(15) $$bfs=\frac{2\cdot pc\cdot r}{\left(r+pc\right)}$$

(16) $$Dice=\frac{2\cdot TP}{2\cdot TP+FP+FN}$$

In this case, True Positives (TPs) are pixels correctly labeled as part of the Region of Interest (RoI). True Negatives (TNs) are pixels that are outside the RoI and have been correctly identified as such. FPs are pixels that are incorrectly classified as part of the RoI when they actually are not. FNs are pixels that are part of the RoI but have not been identified as such in the segmentation. The variable r represents the recall metric, and is defined in (17); pc refers to precision as defined in (18).

(17) $$r=\frac{TP}{TP+FN}$$

(18) $$pc=\frac{TP}{TP+FP}$$

## 3. Results

As a result of this study, three neural networks were trained for the semantic segmentation of structures in laparoscopic cholecystectomy. The implemented architectures were U-Net5ed, SegNet-VGG19, and DeepLabv3+, whose performance was evaluated based on the adjustment of various hyperparameters, as well as by varying relevant parameters for their performance, such as the loss function and activation function, among others. Although not all possible combinations were exhaustively explored, the experiments conducted and presented in this section reflect a representative selection of the trends observed in our data.

In the training of neural networks for semantic segmentation, there may be constraints or preferences regarding the dimensions of the input images. Some backbones tend to have standard input image sizes, especially those pretrained on datasets like ImageNet. However, in general, there is great flexibility in terms of allowable sizes, as long as they are sufficiently large for the convolution and pooling layers to function correctly. Larger input image sizes tend to improve segmentation accuracy, although this implies a higher computational cost. Considering this, the images in the dataset were resized to a resolution of 224 × 224 for U-Net5ed and SegNet-VGG19, while 299 × 299 images were employed for DeepLabv3+. To resize the images, the imresize function of MatLab^®^ was used, applying a bicubic interpolation, which determines the output pixel value as a weighted average of pixels in the nearest 4-by-4 neighborhood.

In the training of neural networks for semantic segmentation, there may be constraints or preferences regarding the dimensions of the input images. Some backbones tend to have standard input image sizes, especially those pretrained on datasets such as ImageNet. However, in general, there is great flexibility in terms of allowable sizes, as long as they are sufficiently large for the convolution and pooling layers to function correctly. Larger input image sizes tend to improve segmentation accuracy, although this implies a higher computational cost. Considering this, the images in the dataset were resized to a resolution of 224 × 224 for U-Net5ed and SegNet-VGG19, while images of 299 × 299 were used for DeepLabv3+. To resize the images, the imresize function of MatLab^®^ was used, applying a bicubic interpolation, which determines the output pixel value as a weighted average of pixels in the nearest 4-by-4 neighborhood.

From the CholecSeg8k dataset, 60% of the images were used for training, and 20% for validation and testing (4848 training images, 1616 validation images, and 1616 test images). When analyzing the characteristics of the dataset, a severe class imbalance becomes evident. Table 1 reflects the distribution of images by class, both in the original dataset and in the training, validation, and test subsets.

Since the objective of the dataset is cholecystectomy surgeries, a higher presence of the classes Liver (present in all images of the dataset) and gallbladder is observed. Additionally, other classes such as Black Background, Fat, Abdominal Wall, Grasper, Gastrointestinal Tract, and L-hook Electrocautery stand out, as they are present in more than 25% of the images. In contrast, there are four classes that appear in less than 10% of the images, which contributes to the class imbalance. In the case of surgical instruments, despite appearing in a considerable number of images, their dimensions are small compared to other elements in the scene, resulting in a low proportion of pixels relative to their number of appearances in the dataset.

**Figure 8:**
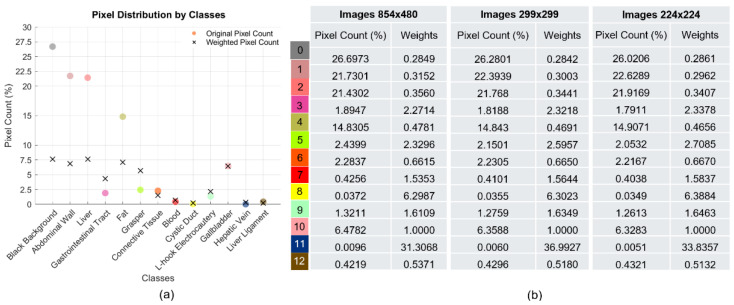
The analysis of dataset characteristics at the pixel level. (**a**) The distribution of pixels by class: circles represent the number of pixels by class before class balancing, and crosses represent the distribution of pixels by class after class balancing. (**b**) Weights applied to balance classes for different dimensions of the images in the dataset.

Figure 8a allows for a more detailed appreciation of the pixel proportions by class (%) in the original dataset, which are represented by circles with the color initially assigned to each class. Four classes account for the highest number of pixels: Black Background represents approximately 27%, Abdominal Wall around 22%, Liver approximately 21%, and Fat about 15% of observations. Additionally, four classes, the blood, cystic duct, hepatic vein, and Liver Ligament, do not reach 1% of pixels in the dataset, as they are small structures with a lower number of occurrences.

Considering the severe class imbalance of the dataset, an algorithm-level class balancing approach was applied, so data augmentation techniques were not used. In this regard, class weighting was performed, and different loss functions that focus on improving the detection of classes with a lower number of observations were employed. Class weighting adjusts the weights during model training to balance the importance of the imbalanced classes, assigning them higher weights. This modifies the loss function to encourage the model to focus more on the minority classes, improving their accuracy. This technique affects the model training process, not the data itself (as oversampling or undersampling would), which makes it especially valuable because it allows addressing class imbalance without manipulating or altering the original dataset, which is desirable when working with medical images.

Figure 8a illustrates to some extent the effect of adjusting the weights in the dataset classes, for which the percentage of pixels by class was multiplied by their respective weights, for the original dataset. The results of this multiplication are indicated by black crosses. This adjustment is perceived as a decrease in the number of pixels for the most represented classes and an increase for the least represented ones, leading to a more balanced distribution. The weights applied for the 13 classes of the dataset are shown in Figure 8b, based on the different dimensions of the images used in the experiment.

To update the learning parameters of the network, all cases were addressed with the SGDM and Adam optimizers. The hyperparameters established are shown in Table 2.

ILR represents the Initial Learning Rate. During the training process, ILR decreased every 10 epochs, reaching a final value of $9.0\times 10^{-5}$ for SGDM, and of $9.0\times 10^{-6}$ for Adam.

### 3.1. Experiment Results

**Figure 9:**
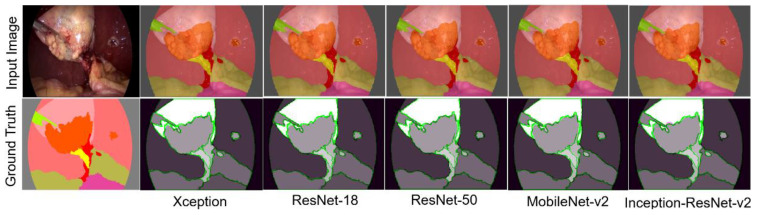
Semantic segmentation obtained with the DeepLabv3+ architecture CE, ReLU, SGDM, and different backbones.

In this study, several experiments were conducted, covering most of the possible combinations based on the variation of activation functions, loss functions, and SGDM and Adam optimizers for each DL architecture. Below, the tests conducted are summarized in three experiments. In the case of the DeepLabv3+ architecture, different pretrained neural networks were used as backbones to compare their behavior. Figure 9 shows the results obtained with the DeepLabv3+ architecture, the ReLU activation function, the CE loss function, and the SGDM optimizer. The backbones used were Xception, ResNet-18, ResNet-50, MobileNet-v2, and Inception-ResNet-v2, respectively.

To facilitate the verification of segmentation accuracy, the images on the right of the ground truth are added. This image was generated by applying the MatLab^®^ function imshowpair (A,B). This function compares two images, A and B, generating an RGB image that overlaps A and B in different color bands. The areas in grayscale indicate where A and B match in intensity, reflecting accurate segmentation. On the other hand, regions in magenta and green highlight differences in intensity and, therefore, represent incorrectly segmented pixels.

In the first column, the upper image is the input image, and the lower image corresponds to the ground truth for the semantic segmentation of the surgical scene. The rest of the images in the upper row show the semantic segmentation obtained by different backbones, while in the lower row, segmentation errors are highlighted in green and magenta. As observed, performance is similar in this image, as is the case with the dataset in general. Regardless of the backbone, the DeepLabv3+ architecture offers positive results.

When analyzing Mean Accuracy (MAcc), better results are obtained with ResNet-18 and ResNet-50, with values of 0.987 and 0.986, respectively. Regarding the Mean IoU (MIoU), the best results are obtained with ResNet-50 and ResNet-18, with values of 0.928 and 0.921, respectively. When calculating the average boundary F1 score (Mbfs), ResNet-50 exhibited the best performance with a value of 0.964. Considering this narrow margin among the results for each case, it was decided to continue with the analysis of the DeepLabv3+ architecture and the ResNet-50 backbone, hereinafter referred to as (DeepLabv3+).

#### 3.1.1. Experiment 1

**Figure 10:**
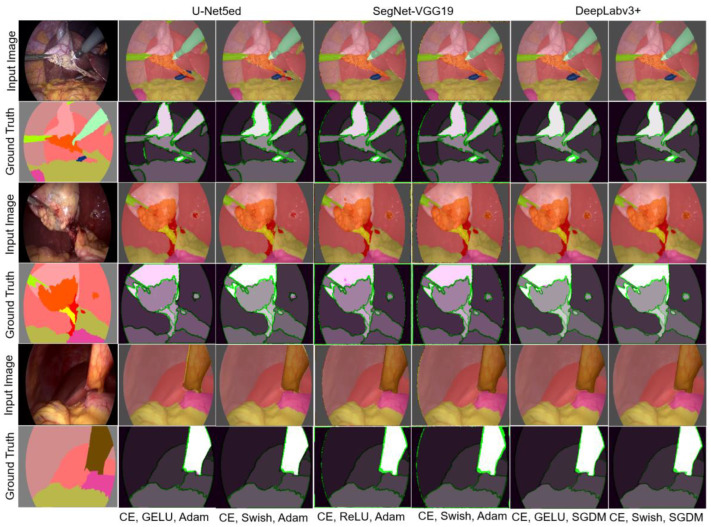
Semantic segmentation obtained with the loss function CE.

The first test was conducted using the CE loss function and the activation functions ReLU, GELU, and Swish. Figure 10 shows the best results obtained in said experiment. The results of the semantic segmentation are presented based on three images, which collectively contain the 13 classes present in the dataset. In all cases, the first column corresponds to these three input images along with their respective ground truth. Subsequently, the results obtained with the U-Net5ed, SegNet-VGG19, and DeepLabv3+ architectures for the SGDM and Adam optimizers are shown.

The SGDM optimizer and DeepLabv3+ was the only architecture that enabled the accurate detection of all the structures present, with the only drawback being some segmentation errors at their borders. In turn, SegNet-VGG19 detects most structures, except for the hepatic vein, which is one of the structures with the fewest annotations in the dataset. Additionally, the U-Net5ed architecture detects all the structures in the image despite the scarce data about some structures; however, it yields a larger number of FPs, which affects recall, accuracy, and precision. This architecture shows a considerable improvement when using Adam, compared with SGDM.

Table 3 presents the general results achieved through the measures of Global Accuracy (GAcc) and MAcc.

An important aspect to consider is the operation of these algorithms during the segmentation of more complex structures that contain fewer occurrences in the dataset and therefore a smaller number of training samples. This is why they are not considered in several studies that employ this dataset. Table 4 summarizes the metrics for the semantic segmentation of structures with the smallest number of annotations. It is observed that the most complex structures to segment are the hepatic vein and cystic duct, obtaining a lower performance by all deep learning architectures. In general, the best performance is obtained with the DeepLabv3+ architecture, the SGDM optimizer, and the Swish and GELU activation functions.

The GELU activation function with the SGDM optimizer improves to some extent semantic segmentation performance compared to the activation function ReLU, which was obvious in the U-Net5ed architecture. Meanwhile, when using Adam, all architectures perform similarly and positively, achieving the segmentation of structures classified as highly complex. Visually speaking, the Swish activation function offered positive results in general. In the case of the SGDM optimizer, the best result was reached with the DeepLabv3+ architecture, while adequate results are obtained with the Adam optimizer for the three architectures. In the case of SegNet-VGG19, difficulties are observed in background and edge segmentation.

The analysis of the general measures indicates that the DeepLabv3+ architecture with the SGDM optimizer obtained the best score most of the time, followed by the SegNet-VGG19 architecture with the Adam optimizer. Regarding qualitative results, the metrics MIoU and Mbfs are considered more representative of the real segmentation result. The U-Net5ed architecture presents the best performance with the Adam optimizer, with the activation function Swish being the most favorable. SegNet-VGG19 improved its performance with Adam, having the highest quantitative results with ReLU and GELU. DeepLabv3+ with SGDM and the activation functions Swish and GELU show the best results. Overall, the DeepLabv3+ architecture, whether with SGDM or with Adam, and the activation functions GELU and Swish, presents the best performance in all scenarios. U-Net5ed was able to detect all the structures, showing significant improvements with the Adam optimizer.

#### 3.1.2. Experiment 2

**Figure 11:**
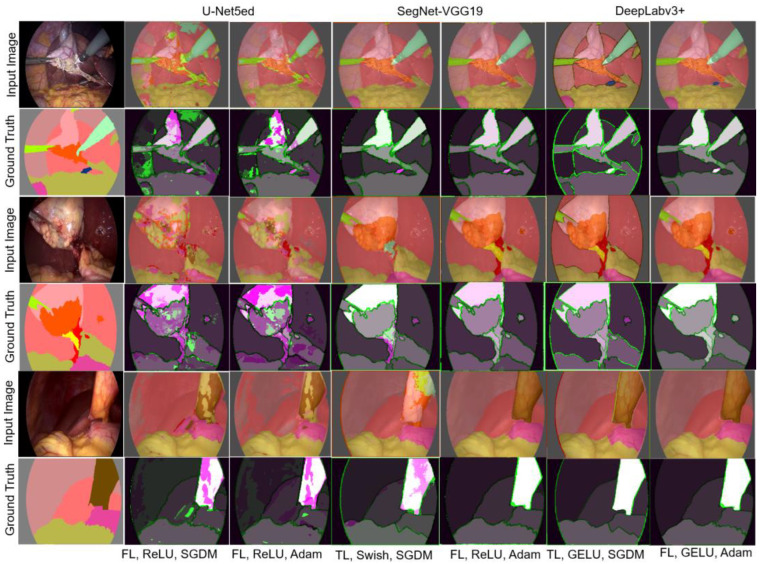
Semantic segmentation obtained with the loss functions FL, DiL, and TL.

The loss functions FL, DiL, and TL were applied and analyzed for each architecture, for the three activation functions under study, and considering the effect of the Adam and SGDM optimizers. In the case of TL, it has been successfully applied in medical images, while FL and DiL, in addition to being suggested for medical images, often report a good performance in the semantic segmentation of structures with a small number of annotations. Figure 11 shows the best results obtained for each architecture in the experiments carried out.

Through a visual inspection of the results, it can be observed that the U-Net5ed network only yielded acceptable results using FL, as it was impossible to segment the hepatic vein and cystic duct structures. A similar behavior was observed with SegNet-VGG19, for which the segmentation of the hepatic vein structure was achieved in all the tested combinations. DeepLabv3+ shows, without a doubt, the best result among the three architectures and allows for detecting all the structures in the dataset by using the activation functions GELU and Swish; however, the reverse is true when ReLU is employed. The results below are the values reached in experiment 2, as shown in Table 5.

The performance of these architectures in the segmentation of structures with a smaller number of observations is verified in Table 6. The FL value was adjusted using $\alpha_{t}=0.25$ for both optimizers; γ=2 and γ=4 were established for SGDM and Adam, respectively.

#### 3.1.3. Experiment 3

**Figure 12:**
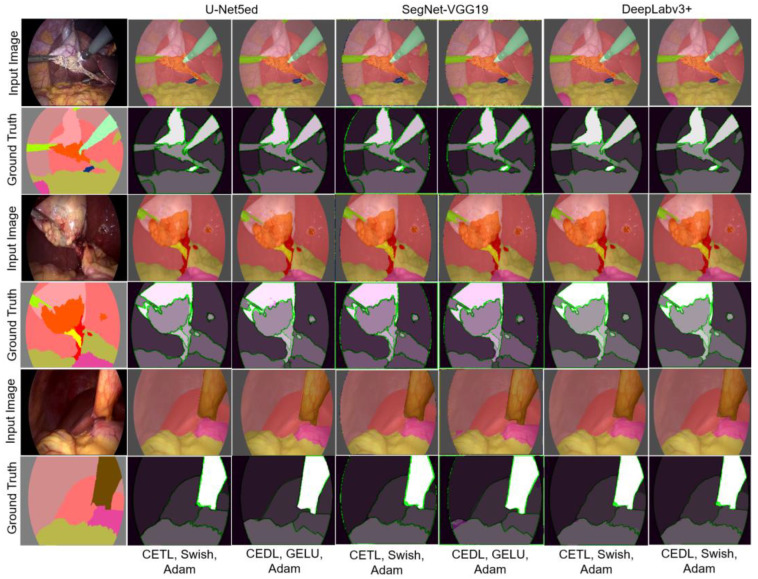
Semantic segmentation obtained with the loss functions CEDL and CETL.

This experiment aims to observe the effect of applying a combination of different loss functions, specifically CEDL and CETL. Tests were conducted with all the activation functions and both optimizers. Due to the scope of the experiment, only the best performance results are shown according to the analyzed loss functions, optimizers, and activation functions. The results are presented in Figure 12.

In this experiment, all structures in the image are accurately detected, and the best results were achieved with the Adam optimizer. When the TL function was used independently, it showed the presence of FPs and, in a smaller proportion, of FNs, mainly on the edges, which is directly related to the parameters adjusted during training. FNs were assigned more weight, with σ=0.3 and β=0.7. When combining TL and CE, results improved; in this case, δ=0.7 was applied, giving more relevance to the CE loss function, and using $Epsilon=1.0\times 10^{-6}$. Table 7 shows the general indicators calculated in this experiment.

According to the values of the general metrics, U-Net5ed can be considered the best performing architecture when combined with Swish and the CETL loss function, and GELU with the CEDL. However, in this case, the visual illustrations serve to highlight how human judgment, based on visual inspection, plays a crucial role in determining the most robust methods, especially in those cases where the metrics are close or identical between different architectures. When analyzing Figure 12, it becomes evident how metrics may not fully reflect the quality of segmentation in situations with correctly identified objects of minority classes or subtle errors in edge delimitation.

The architecture that shows higher precision and exactitude during segmentation is DeepLabv3+, with scarce differences between the segmentation offered by both loss functions. These results are very similar to the results of the DeepLabv3+ architecture combined with the Swish and GELU activation functions, and with the two loss functions analyzed. This analysis can be enriched by verifying the performance of the architectures in the presence of structures with a smaller number of observations. Table 8 reflects that, in terms of accuracy, SegNet-VGG19 exhibits the best performance; however, observing other indicators, U-Net5ed is confirmed as having a better overall performance, followed by DeepLabv3+. It should be noted that FPs are more frequent with the U-Net5ed architecture, which represents an important inconvenience.

**Figure 13:**
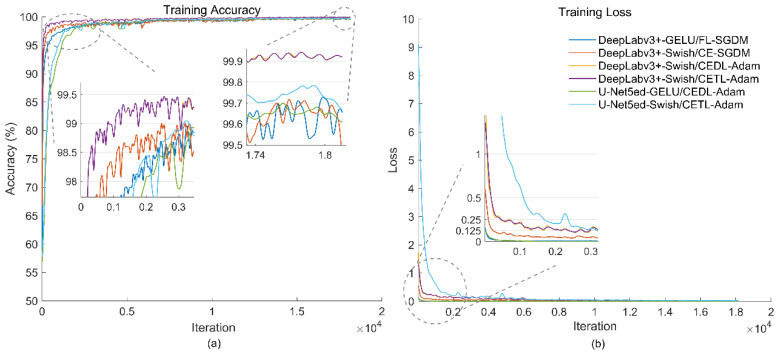
Training curves of the architectures with the best performance. (**a**) Training Accuracy. (**b**) Training Loss.

Figure 13 shows the training curves of the architectures with the best performance in the different experiments: (a) represents the behavior of accuracy, and (b) represents the behavior of the loss functions. The Swish activation function and the DeepLabv3+ architecture are observed to reach high values that are close to the maximum in accuracy, followed by Swish and U-Net5ed. A common denominator among the best performing architectures is the use of combined loss functions with a minimal difference between them in the same DL architecture.

The convergence of all the loss functions shown in the figure exhibits similar loss values and convergence speed. The lowest loss values are obtained with the FL, CEDL, CE, and CETL functions, respectively. Although the loss values reached show some relationship with the accuracy values, they do not determine which architecture will offer the best performance during semantic segmentation. This is observed with FL, which despite reaching high accuracy values on several occasions, and very small loss values during training, results in inefficiency, in most combinations, for the segmentation of structures with a smaller number of observations.

### 3.2. Validation of the Results

To validate the parameters selected in the research, their impact on other DL architectures was evaluated, as well as on another set of medical images. Next, the tests carried out are briefly described.

#### 3.2.1. Behavior of the Selected Parameters in Other DL Architectures

To perform this test, the ninth version of the YOLO (You Only Look Once) architecture [58], recently created and part of a family of neural network architectures for object detection, was selected. However, their use extends to numerous domains. The original YOLOv9 architecture implemented employs the SGDM optimizer, with a momentum of ${9.37\times 10}^{-1}$, ILR of ${1.0\times 10}^{-2}$, and weight decay of ${5.0\times 10}^{-4}$. The activation function used is Sigmoid Linear Unit (SiLU), which corresponds to the configuration of the Swish activation function used in the experiments conducted. The implemented loss function consists of the weighted sum of several individual loss functions, which allows the model to learn to detect objects, classify them, and segment masks with high precision. The combined loss functions are

- Localization Loss $\left(l_{box}\right)$: defined as Complete Intersection over Union (CIoU) loss, and allows for more accurate object detection using bounding boxes (bboxes). This loss not only measures the overlap between predicted and actual bboxes but also considers the distance between their centers, aspect ratio, and scale;
- Objectness Loss $\left(l_{obj}\right)$: defined as Binary Cross Entropy (BCE) loss, and determines whether the bbox contains an object or not;
- Classification Loss $\left(l_{cls}\right)$: defined as BCE loss, and helps the model correctly identify the type of object in each bbox;
- Mask Loss $\left(l_{seg}\right)$: defined as BCE loss with logits (BCEWithLogits), and measures the accuracy with which the predicted segmented masks align with the actual masks.

In addition to these parameters, YOLOv9 introduces techniques such as Programmable Gradient Information (PGI) and Generalized Efficient Layer Aggregation Network (GELAN), which allow for overcoming the loss of information throughout the layers of the network and combining generalized and localized attention to protect crucial information during the detection process, thereby improving accuracy and efficiency in real-time object detection.

Table 9 summarizes the results obtained by this architecture for the semantic segmentation of the surgical scene. The first column shows the result obtained by the original DL architecture and the second column shows the result obtained considering the modification of the parameters proposed in this study; the CETL loss function was implemented as the mask loss $\left(l_{seg}\right)$.

**Figure 14:**
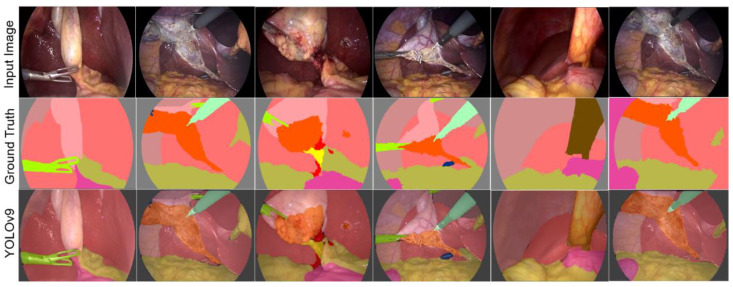
The semantic segmentation of the surgical scene with the YOLOv9 architectures using the parameter configuration proposed in this study.

Figure 14 shows the results offered by the YOLOv9 architecture, using the Adam optimizer, the CETL loss function, and the Swish activation function. The number of epochs was set to 30. As can be observed, the results are satisfactory. It is worth noting that, as in all the tests performed, the results are sensitive to improvements by increasing the number of training epochs, among other parameters. It was verified that the proposed parameters allow improving the performance of this DL architecture.

As observed, the results of YOLOv9 present black color regions that do not belong to a specific class. This may be related to the processing carried out to obtain the semantic segmentation, since this architecture is not originally proposed for this purpose, so it is considered that this aspect can be improved.

#### 3.2.2. Application of the Proposed DL Architecture on Another Dataset

**Figure 15:**
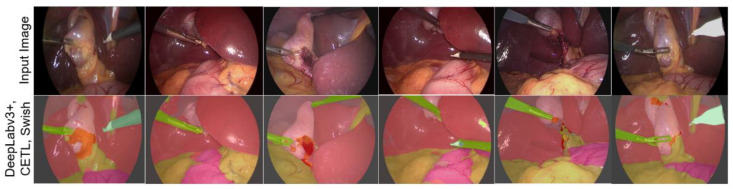
Semantic segmentation with the proposed DL architecture on the Endoscapes dataset.

To verify the generalization and precision capability of the trained DL architecture, an additional dataset was used, and its performance was evaluated through visual inspection, so no metrics were calculated. For this task, the DeepLabv3+ architecture, the CETL loss function, and the Swish activation function were applied. Figure 15 exemplifies some of the results obtained. For this test, the Endoscapes dataset [59] was selected, which contains laparoscopic cholecystectomy videos and has annotations whose purpose is the automated evaluation of the Critical View of Safety (CVS). The images have dimensions of 854 × 480. This dataset has been made public under the CC BY-NC-SA 4.0 license.

Although this dataset does not contain all the structures for which the neural network was trained, it includes several of them such as the Black Background, gallbladder, surgical tools, and cystic duct, among others. Testing the performance of our architecture on this dataset, although it is a risky process because it was not trained with images from it, by containing these structures, it allows verifying the generalization of the proposed architecture and the relevance of scientific research. As can be observed, many of the structures present in the scene are adequately segmented; despite being blurry, it can even be seen that in the first image, a robust segmentation of the structures is performed.

### 3.3. Comparison of Results

Table 10 shows a comparison of the best results obtained in this study with methods developed for the CholecSeg8k dataset. In this case, the data yielded by DeepLabv3+ with the backbone ResNet-50 and the use of the Adam optimizer, the Swish activation function, and the CETL loss function are included. In [31], the results for the classes of the Liver Ligament, blood, cystic duct, and hepatic vein are not reported due to a lack of annotated data.

In [28], the results achieved with the U-Net++ architecture are shown, where the Dice value for the instruments is jointly calculated, denominating this as the general class (Instrument); therefore, the numerical value for the Grasper and L-hook Electrocautery classes is not specified. Classes with a lower number of appearances are grouped into the category Misc, which report a Dice coefficient of 0.16. The Dice value for Instrument is 0.61. The model proposed in [31] is a multi-task encoder based on ResNet-50 and a Multi-Stage Temporal Convolutional Network (MS-TCN).

In [32], a method of Dice-Aware Active Learning based on per-class Uncertainty (DAAL-U) is proposed. In [60], the segmentation corresponding to surgical tools is referred to as (tool) and acquires a Dice coefficient of 0.91. In [61], a Swin-base encoder + Spatial–Temporal Convolutional Network (SP-TCN) architecture is proposed for the semantic segmentation of structures in surgical videos. The evaluation of the results for the CholecSeg8k dataset does not include the structures with a lower number of annotations.

## 4. Discussion

It may be concluded that all architectures allow for the segmentation of structures with a small number of occurrences given a suitable parameter configuration, with the following being determining factors: the optimizer, activation function, and loss function. The results offered by SegNet-VGG19 and U-Net5ed with the Adam optimizer stand out as considerably superior to the values obtained with the SGDM optimizer. DeepLabv3+ exhibited good behavior with both optimizers.

The SegNet-VGG19 architecture achieves good results with combined loss functions; however, the lack of detail in the segmentation of the background, which shows some noise, hinders its performance significantly. In the case of the TL loss function, the variation of the σ and β parameters attempts to remove FPs and/or FNs, and therefore its combination with other loss functions might lead to a superior performance. The experiments conducted proved this hypothesis. The combination of loss functions enabled the improvement in the behavior of all architectures through the Adam optimizer. The result offered by U-Net5ed significantly improves, while DeepLabv3+ enhanced the border segmentation of structures as expected, reducing FNs.

Trainings lasted between 148 and 393 min, and were influenced by factors such as the DL architecture employed, the type of optimizer, and the loss and activation functions. Regarding image segmentation, when using DeepLabv3+ and 299 × 299 images, this takes approximately 90 ms with Adam, and 100 ms with SGDM. In the case of U-Net5ed and SegNet-VGG19, 224 × 224 images are employed, with a segmentation time of 100 ms for U-Net5ed combined with the Adam optimizer, and 90 ms with SGDM. SegNet-VGG19 takes 90 ms with Adam and 56 ms with SGDM.

**Figure 16:**
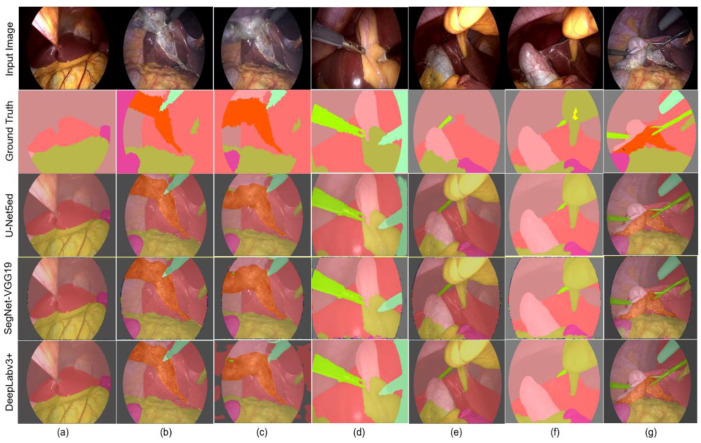
Images with lower performance in semantic segmentation with the CETL, Swish, and Adam parameters. (**a**–**d**) Background labeled as other classes. (**e**) Fat class not labeled. (**f**) Cystic duct structure labeled, but not found in the image. (**g**) Exemplifies cases where the hepatic vein structure is not detected.

When analyzing the images with lower performance in semantic segmentation (IoU values between 0.600 and 0.800), labeling errors were mostly observed in the ground truth of the dataset. Figure 16 shows the results provided by the best performing networks for each DL architecture in different experiments in response to these issues. Despite errors in some ground-truth images, which affected the numerical value of the metrics used to evaluate semantic segmentation, it is observed that the result provided by the trained networks was positive.

Critical cases are presented in Figure 16a–d, where the background is labeled as other classes. In Figure 16e, it is observed that the Fat class was not labeled. In Figure 16f, the presence of the cystic duct structure was labeled, but it is not found in the image. Figure 16g exemplifies the cases where the hepatic vein structure is not detected. As observed in the ground truth, the structure has a very small size, which, together with its low number of appearances in the dataset, determines a low performance. Despite this, the DeepLabv3+ architecture detects it in more than 70% of the images.

### 4.1. Clinical Value of the Research

Laparoscopic cholecystectomy is the gold standard for gallbladder removal; however, this procedure is not exempt from complications. In this sense, the use of semantic segmentation is crucial to accurately identify the elements present in the scene and thus improve the performance of the procedure. Among the fundamental elements to segment in the surgical scene, present in the analyzed dataset, are

- Cystic Duct: Its identification is crucial to avoid bile duct injuries, which is one of the most serious complications of laparoscopic cholecystectomy.
- Gallbladder: Its segmentation allows the surgeon to distinguish it from the surrounding tissues, which is essential for its precise and complete extraction.
- Liver and Liver Bed: The gallbladder rests on them, so their segmentation avoids damage to these tissues during gallbladder dissection.
- Adjacent Tissues and Adhesions: Their segmentation favors adequate dissection and allows avoiding unintentional injuries.
- Surgical Tools: Their segmentation is essential to monitor their position and movement, ensuring that they are used safely and effectively, and allowing the development of computer-assisted surgery applications.

Although the main motivation of this study focuses on favoring the development of applications for robot-assisted cholecystectomy, the findings provide a valuable reference framework for expanding the application of semantic segmentation in various areas of laparoscopic surgery. In laparoscopic nephrectomy, the precise identification of renal anatomy is required to avoid damage to blood vessels and the ureter. On the other hand, in laparoscopic prostatectomy, the correct segmentation of the prostate and surrounding tissues is crucial to minimize complications such as incontinence and erectile dysfunction. The detection of blood accumulation and the presence of gauze is also highly useful.

In general, semantic segmentation enables the identification of critical structures and the delimitation of safe surgical zones, encourages the development of robotic assistance applications for cooperative work between the surgeon and the robot, facilitates the evaluation of the complexity of operations for an effective assignment of surgeries based on the complexity of the surgical procedure and the experience of the surgeons, and, through preprocessing of visual information, improves the visualization of structures hidden by surgical instruments, among other applications [62,63,64].

### 4.2. Key Findings and Limitations

This study focuses on offering more accurate intraoperative segmentation. Table 11 presents the main findings of this research.

An encoder–decoder configuration that improves the performance of the U-Net and SegNet architectures was experimentally determined. Results were obtained with five encoder–decoders in both cases. The U-Net5ed architecture allowed for encoding more detailed and complex features without increasing computational costs, being beneficial for surgical applications that require high precision. The influence of backbones in the SegNet and DeepLabv3+ architectures was evaluated. For SegNet, using VGG19 as the backbone outperformed VGG16 and the original architecture. In DeepLabv3+, ResNet-50 demonstrated the best performance on the dataset. The application of a variable ILR resulted in improvements compared to using a fixed ILR. With a fixed ILR parameter, a reduction of approximately 0.7% is obtained in MAcc during validation.

Regarding the treatment applied to class imbalance, it was experimentally demonstrated that the algorithm-level class balancing approach and the appropriate selection of loss functions improved semantic segmentation performance. In general, it is recommended to avoid the use of oversampling or undersampling techniques when working with medical images, especially if expert supervision is not available. There are also other alternatives such as applying data splitting strategies. It can be said that this adjustment had one of the greatest implications on the final performance of semantic segmentation. The effect of various activation functions and loss functions on semantic segmentation was also compared. It was concluded that GELU and Swish offer better performance than ReLU, and the inclusion of TL in loss functions allowed for adjusting the final results according to the problem under study.

In the presence of class imbalance, the importance of selecting appropriate metrics to evaluate the performance of DL architectures was highlighted, and it was recommended to combine quantitative and qualitative assessments for a more accurate evaluation. The generalization ability and applicability of the proposed architecture were validated through cross-validation on the Endoscape dataset. Additionally, the YOLOv9 architecture provided better performance in semantic segmentation with the Adam optimizer, the Swish activation function, and the CETL loss function, compared to the results obtained with its original configuration on the dataset under study.

Among the limitations of the developed research, the utility of employing multiple additional databases can be mentioned. This would allow for evaluating the robustness and generalization ability of the algorithm in a greater variety of clinical and anatomical scenarios. Experimentation with different attention mechanisms or new DL architectures such as Transformers is also considered appropriate, as well as an in-depth analysis of advanced techniques such as transfer learning and semi-supervised learning, among others. Furthermore, it is considered that minimizing the effects of class imbalance could be enriched through a more detailed analysis, given its relevance in the medical field. Another limitation of this study is the lack of clinical validation, which would enrich the results, providing concrete evidence of the effectiveness and applicability of the findings in real medical applications.

## 5. Conclusions and Future Work

This study addressed the effect of modifying several parameters from different DL architectures for the semantic segmentation of abdominal laparoscopic surgery images. The variation of the activation function and the loss function, among other adjustments, generated promising results. The CEDL and CETL loss functions exhibited the best results, with the application of the class balancing algorithm proving to be adequate. The FL loss function did not show the expected results, noticeably affecting the segmentation of small structures with a small number of observations in all the studied architectures.

The activation function exhibited an important effect on the semantic segmentation results. ReLU often produces acceptable results, while GELU and Swish improve segmentation performance without a significant increase in computational cost. Fine-tuning improved the results offered by the studied architectures. In the case of SegNet-VGG19, it showed better performance than SegNet, with more accurate segmentation. This architecture produces acceptable results and is lighter in terms of both training and segmentation time. Although U-Net5ed offered excellent quantitative results, through visual inspection, it was possible to determine a slightly superior performance of the DeepLabv3+ architecture when using combined loss functions. In general, the use of DeepLabv3+ is suggested, combined with the Adam optimizer, the GELU or Swish activation functions, and the CEDL and CETL loss functions. Similarly, the SGDM optimizer proved to be a good option; however, within the framework of the conducted study, its results were generally inferior.

The tests to validate the performance of the proposed deep learning architecture yielded robust results. In the case of the calculated indicators, accuracy is not as representative of the real results in the presence of class imbalance, which was noticed when visually comparing the results. Therefore, the use of more representative indicators such as IoU, Dice, and bfs is suggested. In this regard, it is important to highlight the qualitative assessment in decision-making to evaluate the performance of algorithms, in conjunction with the calculated metrics and in accordance with the specific task to be performed.

It is noteworthy that several aspects could improve the results obtained, among which are the adjustment of mini-batch size (in this study, good results were obtained with the value 8). The variable ILR of $1.0\times 10^{-3}$ for the SGDM optimizer and of $1.0\times 10^{-4}$ for Adam proved to be adequate. Another hyperparameter that greatly influences the results may be the number of training epochs. In many works consulted, 100, 1000, or more epochs are employed. Additionally, data augmentation can be conducted to alleviate class imbalance and improve training. Emphasis is placed on the usefulness of algorithm-based approaches, taking special care when employing the generation of synthetic images in the medical field. In the case of the loss function, other combinations may improve training performance when paying attention to the concrete aspects of each application.

In future works, we intend to expand the field of study of laparoscopic cholecystectomy to other surgeries under this modality, using a greater variety in the dataset. In addition, advanced techniques such as transfer learning will be explored in depth, and the performance of the proposed parameter configuration will be verified in new architectures, such as Transformers. The study on minimizing the effects of class imbalance will also continue, given its importance in the medical field. The clinical validation of the proposed models represents another significant area of opportunity to ensure their effectiveness and applicability in real medical scenarios, contributing to the development of robot-assisted surgery applications. Addressing these areas will not only mitigate current limitations but also enhance the development of applications for robotic surgery, endowing surgical robots with a certain degree of autonomy.

## Figures and Tables

**Table 1:** Distribution of classes by images in the original dataset (854 × 480).

Class Names	Class Distribution per Image			
	Original	Training Set	Validation Set	Test Set
Black Background	8055 (99.69%)	4836 (99.75%)	1608 (99.50%)	1611 (99.69%)
Abdominal Wall	7255 (89.79%)	4358 (89.89%)	1438 (88.99%)	1459 (90.28%)
Liver	8080 (100%)	4848 (100%)	1616 (100%)	1616 (100%)
Gastrointestinal Tract	4558 (56.41%)	2699 (55.67%)	899 (55.63%)	960 (59.41%)
Fat	7510 (92.95%)	4504 (92.90%)	1511 (93.50%)	1495 (92.51%)
Grasper	6020 (74.50%)	3617 (74.61%)	1204 (74.50%)	1199 (74.20%)
Connective Tissue	1600 (19.80%)	942 (19.43%)	340 (21.04%)	318 (19.68%)
Blood	692 (8.56%)	405 (8.35%)	142 (8.79%)	145 (8.97%)
Cystic Duct	248 (3.07%)	149 (3.07%)	47 (2.91%)	52 (3.22%)
L-hook Electrocautery	2254 (27.90%)	1342 (27.68%)	465 (28.77%)	447 (27.66%)
Gallbladder	6861 (84.91%)	4117 (84.92%)	1372 (84.90%)	1372 (84.90%)
Hepatic Vein	317 (3.92%)	189 (3.90%)	68 (4.21%)	60 (3.71%)
Liver Ligament	240 (2.97%)	146 (3.01%)	55 (3.40%)	39 (2.41%)

**Table 2:** Hyperparameters employed for training.

SGDM		Adam	
Epochs	30	Epochs	30
Initial Learning Rate	$1.0\times 10^{-3}$	Initial Learning Rate	$1.0\times 10^{-4}$
Mini-Batch Size	8	Mini-Batch Size	8
Weight Decay	$5.0\times 10^{-3}$	Weight Decay	$5.0\times 10^{-3}$
Momentum	$9.0\times 10^{-1}$	Gradient Decay Factor ($\beta_{1}$)	$9.0\times 10^{-1}$
		Square Gradient Decay Factor ($\beta_{2}$)	$9.99\times 10^{-1}$
		Epsilon	$1.0\times 10^{-8}$

**Table 3:** Metrics obtained with the parameters of experiment 1.

DL Architecture	Activation Function	Loss Function/Optimizer	GAcc	MAcc	MIoU	Mbfs
U-Net5ed	GELU	CE/Adam	0.987	0.977	0.862	0.919
	Swish	CE/Adam	0.988	0.979	0.882	0.922
SegNet-VGG19	ReLU	CE/Adam	**0.993**	**0.988**	0.927	0.961
	Swish	CE/Adam	0.992	**0.988**	0.903	0.953
DeepLabv3+	GELU	CE/SGDM	**0.993**	0.987	0.916	0.964
	Swish	CE/SGDM	0.992	0.986	**0.932**	**0.965**

Best performance is denoted in bold.

**Table 4:** Metrics for semantic segmentation of structures with the smallest number of annotations in experiment 1.

Structure	Metric	U-Net5ed		SegNet-VGG19		DeepLabv3+	
		GELU/CE Adam	Swish/CE Adam	ReLU/CE Adam	Swish/CE Adam	GELU/CE SGDM	Swish/CE SGDM
Blood	Acc	0.940	0.944	0.943	0.939	**0.949**	0.947
	IoU	0.730	0.751	0.653	0.830	0.864	**0.867**
	Mbfs	0.669	0.699	0.851	0.824	0.905	**0.908**
Cystic Duct	Acc	0.968	0.972	0.986	**0.991**	0.976	0.979
	IoU	0.443	0.712	0.843	0.689	**0.865**	0.849
	Mbfs	0.859	0.838	0.885	0.852	**0.949**	0.842
Hepatic Vein	Acc	0.993	0.988	0.997	0.998	**0.999**	0.993
	IoU	0.440	0.408	0.587	0.497	0.402	**0.627**
	Mbfs	0.577	0.539	0.637	0.628	0.538	**0.655**
Liver Ligament	Acc	0.991	0.990	**0.999**	**0.999**	**0.999**	**0.999**
	IoU	0.987	0.980	0.978	0.966	**0.995**	**0.995**
	Mbfs	**0.974**	0.957	0.871	0.836	0.965	0.970

Best performance is denoted in bold.

**Table 5:** Metrics obtained with the parameters of experiment 2.

DL Architecture	Activation Function	Loss Function/Optimizer	GAcc	MAcc	MIoU	Mbfs
U-Net5ed	ReLU	FL/SGDM	0.733	0.516	0.361	0.470
	ReLU	FL/Adam	0.828	0.558	0.466	0.548
SegNet-VGG19	Swish	TL/SGDM	0.981	0.677	0.646	0.950
	ReLU	FL/Adam	0.992	0.903	0.877	0.959
DeepLabv3+	GELU	TL/SGDM	0.990	**0.969**	0.913	**0.975**
	GELU	FL/Adam	**0.993**	0.946	**0.929**	0.968

Best performance is denoted in bold.

**Table 6:** Metrics for semantic segmentation of structures with the smallest number of annotations in experiment 2.

Structure	Metric	U-Net5ed		SegNet-VGG19		DeepLabv3+	
		ReLU/FL SGDM	ReLU/FL Adam	Swish/TL SGDM	ReLU/FL Adam	GELU/TL SGDM	Swish/FL Adam
Blood	Acc	0.367	0.453	0	0.920	0.930	**0.932**
	IoU	0.239	0.365	0	0.823	**0.872**	0.865
	Mbfs	0.403	0.502	0	0.798	**0.913**	0.903
Cystic Duct	Acc	0	0	0	**0.968**	0.942	0.917
	IoU	0	0	0	0.836	**0.901**	0.862
	Mbfs	0	0	0	0.905	**0.968**	0.939
Hepatic Vein	Acc	0	0	0	0	**0.887**	0.558
	IoU	0	0	0	0	**0.852**	0.558
	Mbfs	0	0	0	0	**0.944**	0.856
Liver Ligament	Acc	0.511	0.597	0	**0.999**	0.995	**0.999**
	IoU	0.328	0.463	0	0.993	0.471	**0.996**
	Mbfs	0.463	0.499	0	0.958	**0.989**	0.978

Best performance is denoted in bold.

**Table 7:** Metrics obtained with the parameters of experiment 3.

DL Architecture	Activation Function	Loss Function/Optimizer	GAcc	MAcc	MIoU	Mbfs
U-Net5ed	Swish	CETL/Adam	0.994	0.978	0.959	**0.973**
	GELU	CEDL/Adam	**0.995**	0.977	0.957	**0.973**
SegNet-VGG19	Swish	CETL/Adam	0.994	**0.984**	0.926	0.969
	GELU	CEDL/Adam	0.994	0.983	0.947	0.969
DeepLabv3+	Swish	CETL/Adam	0.993	0.976	**0.977**	0.972
	Swish	CEDL/Adam	0.994	0.979	0.957	0.972

Best performance is denoted in bold.

**Table 8:** Metrics for semantic segmentation of structures with the smallest number of annotations in experiment 3.

Structure	Metric	U-Net5ed		SegNet-VGG19		DeepLabv3+	
		Swish/CETL Adam	ReLU/CEDL Adam	Swish/CETL Adam	GELU/CEDL Adam	Swish/CETL Adam	Swish/CEDL Adam
Blood	Acc	0.942	**0.951**	0.942	0.940	0.940	0.943
	IoU	0.885	0.883	0.887	0.880	**0.935**	0.881
	Mbfs	0.886	0.883	0.878	0.882	**0.917**	**0.917**
Cystic Duct	Acc	0.948	0.964	0.972	**0.977**	0.957	0.954
	IoU	0.898	0.915	0.870	0.873	**0.958**	0.906
	Mbfs	0.954	0.958	0.927	0.925	0.968	**0.969**
Hepatic Vein	Acc	0.919	0.881	**0.963**	0.948	0.897	0.928
	IoU	0.856	0.819	0.486	0.748	**0.917**	0.849
	Mbfs	0.927	**0.928**	0.737	0.875	0.924	0.905
Liver Ligament	Acc	**0.999**	0.998	**0.999**	**0.999**	**0.999**	**0.999**
	IoU	0.998	0.997	0.994	0.993	**0.999**	0.997
	Mbfs	**0.992**	0.989	0.961	0.962	0.984	0.981

Best performance is denoted in bold.

**Table 9:** Comparison of semantic segmentation in the YOLOv9 architecture with the proposed parameters (Adam, Swish, and CETL).

Class Names	YOLOv9	
	Original	Ours
Black Background	0.752	**0.831**
Abdominal Wall	0.860	**0.865**
Liver	0.855	**0.864**
Gastrointestinal Tract	0.705	**0.735**
Fat	0.780	**0.801**
Grasper	0.652	**0.670**
Connective Tissue	0.752	**0.776**
Blood	0.536	**0.557**
Cystic Duct	0.534	**0.542**
L-hook Electrocautery	0.649	**0.793**
Gallbladder	0.802	**0.819**
Hepatic Vein	0.361	**0.464**
Liver Ligament	0.879	**0.887**

Best performance is denoted in bold.

**Table 10:** Comparison between the results obtained (DeepLabv3+-ResNet-50, CETL, Swish) and the literature.

Structure	Dice				IoU			
	U-Net++ [28]	[31]	SegGPT+ SAM [60]	Ours	DAAL-U [32]	Swin base + SP-TCN [61]	YOLOv9 *	Ours
Black Background	0.98	0.964	0.98	**0.996**	0.954	0.974	0.831	**0.997**
Abdominal Wall	0.83	0.574	0.84	**0.995**	0.827	0.741	0.865	**0.994**
Liver	0.75	0.796	0.90	**0.991**	0.708	0.775	0.864	**0.992**
Gastrointestinal Tract	0.11	0.593	0.95	**0.972**	0.093	0.571	0.735	**0.976**
Fat	0.91	0.534	0.84	**0.994**	0.828	0.842	0.801	**0.994**
Grasper	Instrument	0.536	---	**0.989**	0.404	0.735	0.670	**0.993**
Connective Tissue	Misc	0.340	**0.99**	0.976	0.396	0.312	0.776	**0.978**
Blood	Misc	---	**0.98**	0.929	---	---	0.557	**0.935**
Cystic Duct	Misc	---	---	**0.918**	0	---	0.542	**0.958**
L-hook Electrocautery	Instrument	0.653	---	**0.980**	0.401	0.682	0.793	**0.991**
Gallbladder	0.63	0.741	0.93	**0.974**	0.542	0.611	0.819	**0.985**
Hepatic Vein	Misc	---	---	**0.627**	---	---	0.464	**0.917**
Liver Ligament	Misc	---	---	**0.995**	---	---	0.887	**0.999**

Best performance is denoted in bold, ---: Not reported, * DL architecture trained in this research.

**Table 11:** Summary of key findings of this research.

Key Findings	Details
Proposed Parameter Configuration	A five-encoder–decoder configuration is proposed to improve the performance of the U-Net architecture (U-Net5ed).
	The SegNet-VGG19 and DeepLabv3+-ResNet-50 architectures are implemented to improve performance.
	A variable ILR of $1.0\times 10^{-3}$ for SGDM and $1.0\times 10^{-4}$ for Adam is configured.
Effective Approach to Class Imbalance	The application of class balance improved MAcc by approximately 8.05% with DeepLabv3+, ReLU, CE, and the SGDM optimizer compared to the same configuration without class balancing.
	The implementation of the Swish activation function is proposed.
	The CETL loss function is proposed.
	Appropriate metrics are proposed to assess the performance of DL architectures in the presence of class imbalance. The proposed parameter configuration considers quantitative and qualitative assessments.
Improvement in DL Architectures	Parameter configurations are achieved that enable high success rates in semantic segmentation for all studied DL architectures.
	The DeepLabv3+-ResNet-50 architecture, with CETL and Swish, offers the best overall results in semantic segmentation among the consulted literature.
	Adaptation of the YOLOv9 architecture for semantic segmentation of the surgical scene and improvement in its performance compared to the original configuration.

## Data Availability

Data are contained within the article.
